# Editorial: Root functional traits: From fine root to community-level variation

**DOI:** 10.3389/fpls.2023.1152174

**Published:** 2023-02-15

**Authors:** Enrique G. de la Riva, Kira Borden, Ivika Ostonen, Patompong Saengwilai, Iván Prieto

**Affiliations:** ^1^ Ecology Department, Faculty of Biology and Environmental Sciences, Universidad de León, León, Spain; ^2^ School of the Environment, Trent University, Peterborough, ON, Canada; ^3^ Institute of Ecology and Earth Sciences, University of Tartu, Tartu, Estonia; ^4^ Department of Biology, Faculty of Science, Mahidol University, Bangkok, Thailand

**Keywords:** mycorrhizal, economic spectrum, root plasticity, specific root lenght, ecosystem service

## Introduction

Plant roots perform multiple essential functions defining plant ecological success and ecosystem functioning. For instance, roots are vital for plant nutrient and water uptake, thus regulating net primary production and nutrient cycling (Freschet et al., 2021). In the last decade, the adoption and advancement of a functional trait approach has greatly improved our understanding of root ecology, evidenced by the recent increase of global syntheses on root trait research (e.g., Freschet et al., 2018; Bergmann et al., 2020; Carmona et al., 2021; Freschet et al., 2021). However, there are still gaps and controversy in our understanding of root trait–functioning relationships (Freschet et al., 2021). Roots display a wide diversity of morphologies and symbiotic associations (i.e., with mycorrhizal fungi and rhizobium), which has made it challenging to seek general patterns across the diverse taxa that inhabit different ecological conditions worldwide (Ma et al., 2018). In this special issue, we bring together studies on root ecology that tackle important unresolved questions and emerging topics, which collectively highlight new knowledge and critical knowledge gaps in belowground ecology.

## Root hydraulic redistribution

Water relations are key to understanding the ecology of terrestrial plant communities, and one determining component of water balance is the process of hydraulic redistribution (HR; Caldwell et al., 1998). The term refers to the passive movement of water through plant roots from moist to dry soil layers following a water potential gradient (Prieto et al., 2012). Many factors, such as plant transpiration (Howard et al., 2009), root architecture (Scholz et al., 2008) and soil conditions, e.g. soil humidity and texture (Prieto et al., 2010) affect the direction and magnitude of HR that has been found to be highly variable across species and ecosystems worldwide (Neumann and Cardon, 2012). In a comprehensive review in this special issue, Yang et al. put forward that plant characteristics such as plant transpiration and root length were the main determinants of HR magnitude whereas soil factors such as water table depth or soil texture were also important yet indirect drivers.

## Root morphological traits and resource uptake strategies

The ability of plants to acquire soil water and nutrients determines their competitive success and productivity (Erktan et al., 2018a). In this regard, the variation in root traits in terms of resource acquisition has been gaining research attention (Freschet et al., 2021). Previous efforts to understand how functional traits are organized across terrestrial plants have revealed the existence of an acquisition – conservation trade-off, known as the leaf economic spectrum (Wright et al., 2004), but whether such a high degree of organization is also seen in root traits remains controversial and may depend on the type of mycorrhizal association (e.g., Bergmann et al., 2020; de la Riva et al., 2021). In this issue, An et al. and de la Riva et al. demonstrate that there is a main trend of variation in the multidimensional root space in line with expectations of the root economics spectrum in over 300 species in China and Spain, whereas Jiang et al. found weak or no correlation between fine-root traits in 48 species from a single semiarid ecosystem. Further, Wang et al. provide insights into the existence of a trade-off between the number and the size of fine roots of temperate tree species. Collectively these results suggest belowground trait covariation and trade-offs are strongly driven by environmental gradients (de la Riva et al., 2016; Erktan et al., 2018b) and capture different plant strategies to a wide range of environmental conditions (Díaz et al., 2016) that may be in response to a trade-off between growth and survival (Reich, 2014).

## Root plasticity

Phenotypic variation is an important driver of plant performance under different environmental conditions (Cabal et al.). The majority of research on phenotypic variation to date has focused aboveground (e.g., Roscher et al., 2018; Valladares et al., 2002) and we are still far from understanding the full extent to which environmental conditions elicit a plastic response belowground (Freschet et al., 2021). This special issue brings an essential milestone to this research frontier by gathering several empirical studies showing a rapid plastic response of root traits under changing environmental conditions. Fratte et al. demonstrate that mulching favored the establishment of plant communities with lower plasticity through an adaptive convergence between analogous traits at leaf and root levels. Using a multi-generation experiment with an annual herb (*Papaver rhoeas*), March-Salas et al. proved that precipitation predictability promotes intra- and trans-generational plasticity in root traits, observing differential root trait responses between ancestors and descendants. Moreover, Xu et al. found that, for lianas and vines in tropical ecosystems, phenotypic variation in root diameter in root tips is strongly linked to changes in cortex thickness and cortex cell size rather than on stele diameter variation. These studies widen the characterisation of trait phenotypic variability and decipher the complex and context dependent interactions between root traits and the environment.

## Root and soil interactions

Root-soil interactions occur at multiple spatial and temporal scales and are driven by complex processes occurring between roots, microbes, and the soil environment. Discerning relationships between root traits and root-soil interactions can improve our understanding of plant responses to, and their effects on, the environment (Violle et al., 2007). Song et al. and Borden et al. conduct detailed measurements of rhizosphere enzymatic activity and root-rhizosphere respiration rates, respectively. Both studies consider rhizosphere activity as the result of direct root activity and indirect effects of roots on microbial activity. Song et al. found that the spatial distribution of enzyme activity along the root growth axis was associated with larger root diameter in European beech (*Fagus sylvatica*), and longer root hair length in Norway spruce; and contrasting distributions with distance from rhizoplane suggesting differential contributions of root vs. microbial enzymatic activity. In a field study, Borden et al. found specific root respiration covaried with morphological and chemical root traits, and while microbial abundance in the rhizosphere coordinated with root trait variation, this was not the case in bulk soil.

## Ecosystem functioning and services

Theoretical and empirical evidence predicts that root traits are directly linked with soil structure, nutrient cycling, production and, consequently, ecosystem functioning (Freschet et al., 2021). Thus, roots influence the levels of productive and regulatory ecosystem functions and might directly affect ecosystem services (Freschet et al., 2021), but, as Wang et al. stated in this special issue: “*the importance of root traits in ecosystem-level functioning, is increasingly recognized but still not well-understood*”. This special issue showcases studies observing the role of root traits as key drivers of ecosystem services, such as phytoremediation, crop productivity and carbon cycle. Fratte et al. demonstrate that mulching application increases below-ground biomass, which may favour the proliferation of microbes devoted to soil organic contaminants’ degradation. In another study with cropped lentil plants in arid nutrient-poor environments, El-hady et al. showed that the application of root activator and phosphorus enhanced plant growth and productivity by invigorating root traits. While Borden et al. identify connections between root trait variation with carbon dioxide emissions from soil. These studies improve our understanding of the direct benefits of plant root systems in delivery of ecosystem services *via* root trait-function relationships.

## Conclusions

This special issue brings together research from multiple fields of root ecology that are unified in their trait based approach. Taken together, this special issue gives a complex but realistic picture of the multidimensional and dynamic roles roots play belowground, defining plant resource uptake strategies and performance, and driving ecosystem functioning and services. However, the ability to scale up fine root trait variation to community and ecosystem functioning level requires more critical investigations that make empirical connections between anatomical, morphological, and physiological characteristics of plant roots with ecosystem scale processes.

## Author contributions

All authors listed have made a substantial, direct, and intellectual contribution to the work and approved it for publication.
